# Effectiveness of adalimumab in treating patients with active psoriatic arthritis and predictors of good clinical responses for arthritis, skin and nail lesions

**DOI:** 10.1136/ard.2009.111856

**Published:** 2009-10-07

**Authors:** F Van den Bosch, B Manger, P Goupille, N McHugh, E Rødevand, P Holck, R F van Vollenhoven, M Leirisalo-Repo, O FitzGerald, M Kron, M Frank, S Kary, H Kupper

**Affiliations:** 1 University Hospital, Ghent, Belgium; 2 Universität Erlangen/Nürnberg, Erlangen, Germany; 3 Université François Rabelais, Tours, France; 4 Royal National Hospital, Bath, UK; 5 St Olavs Hospital, Trondheim, Norway; 6 Regionshospitalet Silkeborg, Silkeborg, Denmark; 7 The Karolinska Institute, Stockholm, Sweden; 8 University Central Hospital, Helsinki, Finland; 9 St Vincent’s University Hospital, Dublin, Ireland; 10 Abbott GmbH & Co KG, Ludwigshafen, Germany

## Abstract

**Objectives:**

To evaluate the effectiveness of adalimumab in patients with psoriatic arthritis (PsA) and identify predictors of good clinical response for joint and skin lesions.

**Methods:**

Patients received adalimumab 40 mg every other week in addition to standard therapy in this prospective, 12-week, open-label, uncontrolled study. Four definitions of good clinical response were used: ⩾50% improvement in American College of Rheumatology response criteria (ACR50), good response according to European League Against Rheumatism (EULAR) guidelines, a ⩾3-grade improvement in Physician Global Assessment of psoriasis (PGA) and a ⩾50% improvement in the Nail Psoriasis Severity Index (NAPSI). Response predictors were determined by logistic regression with backward elimination (selection level was 5%).

**Results:**

Of 442 patients, 94% completed 12 weeks of treatment. At week 12, 74%, 51% and 32% of the patients had achieved ACR20, 50 and 70, respectively; 87% and 61% experienced moderate and good responses according to EULAR criteria, respectively. The percentage of patients with PGA results of “clear/almost clear” increased from 34% (baseline) to 68%. The mean NAPSI score was reduced by 44%. No new safety signals were detected. A lower Health Assessment Questionnaire Disability Index (HAQ-DI) score, greater pain assessment, male sex and absence of systemic glucocorticoid therapy were strongly associated with achievement of ACR50 and good response according to EULAR criteria. In addition, greater C-reactive protein concentration and polyarthritis predicted ACR50, and non-involvement of large joints predicted a good response according to EULAR criteria.

**Conclusions:**

Adalimumab was effective in patients with PsA. Lower impairment of physical function, greater pain, male sex and no systemic treatment with glucocorticoids were factors that increased the chance of achieving a good clinical response.

Few studies have addressed whether predictors for a good clinical response to treatment with tumour necrosis factor (TNF) antagonists in patients with psoriatic arthritis (PsA) can be identified. The STEREO (for “SafeTy and Efficacy of adalimumab in patients with active psoriatic arthritis – an open-label, multinational study to evaluate the Response to Every-Other week adalimumab when added to insufficient standard therapy including patients who failed prior treatment with other TNF inhibitors”) trial prospectively evaluated the treatment effect of adalimumab in >400 patients with active PsA who were eligible for treatment with TNF antagonists in daily rheumatology practice.^1^ ^2^ We also evaluated predictive factors for a good clinical response to adalimumab with respect to arthritis, skin and nail disease.

## Methods

### Patients

Main inclusion criteria were: age ⩾18 years, PsA diagnosed by a rheumatologist, ⩾3 tender and ⩾3 swollen joints, previous treatment with ⩾1 disease-modifying antirheumatic drugs (DMARDs) and enrolment in accordance with each participating country’s current guidelines for anti-TNF treatment of PsA. DMARDs, non-steroidal anti-inflammatory drugs (NSAIDs), or oral glucocorticoids (⩽10 mg prednisolone equivalent/day), as well as topical psoriasis therapy, could be continued if the dosage was stable (additional details regarding the inclusion/exclusion criteria and study design are available in the supplementary material).

### Study design and measures

STEREO was a prospective, open-label, uncontrolled study conducted in nine European countries. Patients self-administered adalimumab 40 mg (Abbott Laboratories, Abbott Park, Illinois, USA) subcutaneously every other week for 12 weeks in addition to their pre-existing antirheumatic treatment. Patients who benefited from adalimumab therapy could continue up to week 20 if adalimumab was not commercially available. Observed data at week 12 were used for all effectiveness analyses. Presence or absence of dactylitis, defined as swelling of the entire finger or toe, and enthesitis at the heels were documented at baseline. Measures of effectiveness for PsA were at least 20%, 50% and 70% improvements in the American College of Rheumatology response criteria (ACR20, ACR50 and ACR70, respectively),^3^ tender joint count (TJC; 0–78 joints), swollen joint count (SJC; 0–76 joints), the 28-joint Disease Activity Score (DAS28) based on erythrocyte sedimentation rate (ESR; mm/first hour),^4^ moderate and good European League Against Rheumatism (EULAR) response criteria using the DAS28,^5^ and the PsA response criteria (PsARC),^6^ which was modified by using a 0–100 mm visual analogue scale (VAS) for the Physician or Patient Global Assessment of disease activity (PhGA or PaGA). Additional measurements included 0–100 mm VAS for pain, the Health Assessment Questionnaire Disability Index (HAQ-DI; score of 0–3),^7^ and C-reactive protein (CRP) concentration (mg/dl). Psoriasis was assessed by Physician Global Assessment (PGA) for psoriasis (a 7-point scale ranging from “clear” to “severe”),^8^ target lesion assessment (total plaque score 0–15, not assessed at week 2) requiring a lesion of ⩾2 cm in the greatest diameter at baseline,^8^ Nail Psoriasis Severity Index (NAPSI; 0–80, only of the hands),^9^ and the Dermatology Life Quality Index (DLQI; score of 0–30).^10^ NAPSI and DLQI were evaluated only at baseline, week 12 and week 20.

### Statistical analysis

All patients who received at least one adalimumab injection were included in the analyses. Endpoints for good clinical responses at week 12 were achievement of ACR50, a good response according to EULAR criteria for PsA,^1^ ^11^ ^12^ improvement by ⩾3 grades in PGA for psoriasis (evaluated in patients with PGA worse than “mild” at baseline) and a ⩾50% improvement in NAPSI score for psoriatic nail disorder (evaluated in patients with NAPSI ⩾10 at baseline).

Continuous variables analysed as possible predictors of good clinical response for joint and skin/nail manifestations were age (per year), duration of PsA (per year), CRP (per mg/dl), PhGA and PaGA (per mm), pain (per mm); and DAS28 (per unit). In addition, HAQ-DI score (per unit) was evaluated for PsA, and duration of psoriasis (per year) and DLQI score (per unit) were evaluated for skin and nail disorders.

Categorical variables (yes vs no) analysed as possible predictors of good clinical response for joint and skin/nail manifestations were: male sex; dactylitis; enthesitis; prior TNF antagonist therapy; ongoing systemic treatment with ⩾1 DMARDs, with sulfasalazine (SSZ), or with glucocorticoids; history of tobacco use; ⩾1 inflamed large joint (knee, shoulder, elbow, hip); prior treatment with >2 DMARDs; polyarthritis (⩾5 swollen joints) versus oligoarthritis (<5 swollen joints); and PGA >“moderate” versus PGA ⩽“moderate”. For skin/nail manifestations only, ongoing topical treatment with glucocorticoids and prior ultraviolet A (UVA) and/or psoralen and UVA (PUVA) phototherapy were evaluated.

Crude odds ratios (ORs) with 95% confidence intervals (CIs) and p values based on two-sided χ^2^ tests (continuous variables) or Fisher exact tests (categorical variables) were calculated for all possible predictive factors. For the identification of predictors of ACR50 and good responses according to EULAR criteria, the ensemble of all factors was investigated by logistic regression with backward elimination (selection level was 5%). The predictive value of the final model was assessed by calculating the area under the receiver operating characteristic (ROC) curve. For a ⩾3-grade improvement in PGA and a ⩾50% improvement in NAPSI score, we separately investigated only the predictors because datasets for the complete ensemble of possible predictors were missing in >20% of patients with skin and nail lesions; thus, a selection bias could not be excluded. All values presented are mean (SD) unless otherwise noted.

## Results

### Patient disposition, withdrawals and adalimumab treatment duration

Of the 442 patients enrolled, 94% completed week 12 and 39% continued beyond week 12. During the complete treatment period, 6 (1.4%) patients withdrew because of unsatisfactory therapeutic response and 26 (5.9%) patients withdrew because of adverse events. Other reasons (withdrawal of consent, protocol violation, loss to follow-up, or other) are not shown. The mean adalimumab treatment duration was 103 days (median, 85 days).

### Patient characteristics at baseline

Patient baseline characteristics are shown in table 1. All patients had psoriatic symptoms at baseline and/or a history of psoriasis. Plaque psoriasis (defined as PGA not “clear”) was documented in 366 (82.8%) patients, encompassing 206 patients with PGA greater than “mild” (“mild to moderate” in 59 patients, “moderate” in 82 patients, “moderate to severe” in 46 patients and “severe” in 19 patients). Psoriatic nail dystrophy (NAPSI >0) was reported for 259 (59.3%) patients, including 164 patients with a mean NAPSI of ⩾10.

**Table 1:** Baseline characteristics of patients with psoriatic arthritis (PsA) at baseline

	All patients (n = 442)
Female, n (%)	221 (50.0)
Median (SD) age, years	47.8 (11.5)
Mean (SD) PsA duration, years	10.6 (8.2)
Mean (SD) psoriasis duration, years	19.4 (12.9)
Rheumatoid factor positive, n (%)	51 (11.6)
Anti-CCP antibody positive, n (%)	28 (6.3)
HLA-B27 positive, n (%)	102 (23.3)
Dactylitis, n (%)	131 (29.7)
Enthesitis, n (%)	154 (34.9)
Swollen joint count (0–66), median (quartile 1, quartile 3)	8 (5, 13)
Mean (SD) DAS28	4.93 (1.15)
Prior anti-TNF therapy, n (%)	66 (14.9)
Ongoing DMARD therapy, n (%)	301 (68.1)
Ongoing systemic glucocorticoid therapy*, n (%)	128 (28.9)

*Maximum, prednisone equivalent 10 mg/day.

CCP, cyclic citrullinated peptide; DAS28, 28-joint Disease Activity Score; DMARD, disease-modifying antirheumatic drug; HLA, human leukocyte antigen; TNF, tumour necrosis factor.

### Effectiveness of adalimumab treatment

**Figure 1:**
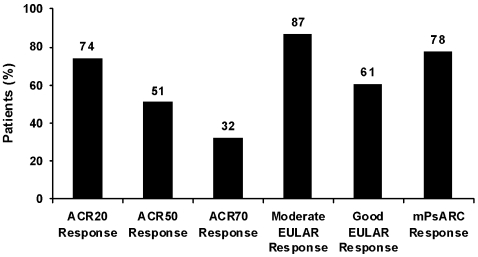
Percentages of patients achieving American College of Rheumatology 20%, 50% and 70% response criteria (ACR20/50/70), moderate and good European League Against Rheumatism (EULAR) criteria responses and the modified psoriatic arthritis response criteria (mPsARC) at week 12 (n = 414). Data are from observed cases.

**Figure 2:**
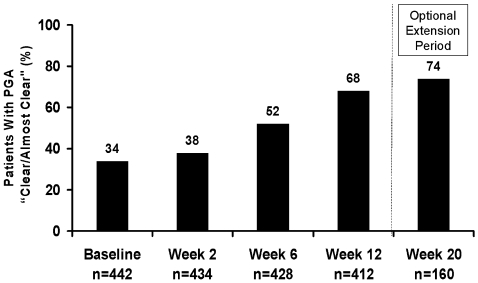
Percentages of patients achieving Physician Global Assessment of psoriasis (PGA) “clear” or “almost clear” up to week 20. All patients are included, data are from observed cases.

**Figure 3:**
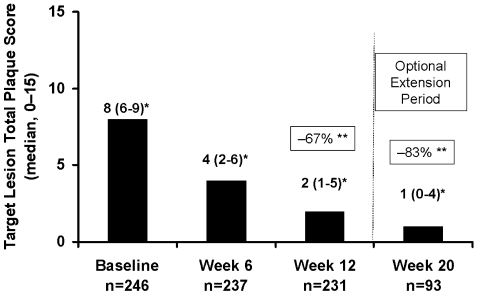
Improvement in median target lesion total plaque score up to week 20 for patients with target lesion ⩾2 cm in greatest diameter at baseline. Data are from observed cases. *Quartile 1 to quartile 3. **Median change (means not presented because of skewed distributions).

**Figure 4:**
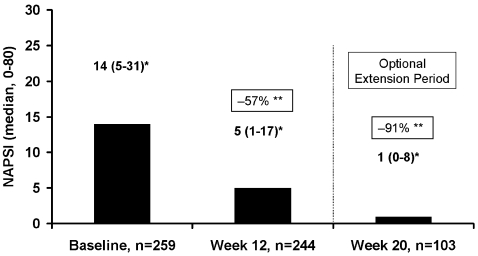
Improvement in median Nail Psoriasis Severity Index (NAPSI) score at weeks 12 and 20 for patients with nail dystrophy (NAPSI >0) at baseline. Data are from observed cases. *Quartile 1 to quartile 3. **Median change (means not presented because of skewed distributions).

ACR, EULAR and modified PsARC response rates at week 12, percentages of patients with PGA “clear” or “almost clear” and changes in target lesion and NAPSI score are shown in figs 1–4. The mean number of tender and swollen joints and mean DAS28, HAQ-DI and DLQI scores were reduced by 61%, 73%, 45%, 47% and 41%, respectively, from baseline to week 12. The supplementary material provides a detailed summary of effectiveness measures.

### Identification of predictors of a good clinical response

#### Predictors of good clinical response for PsA at week 12

The logistic regression with backward elimination revealed that a lower HAQ-DI score, male sex, no systemic therapy with glucocorticoids and a greater patient’s assessment of pain were strongly associated with achievement of ACR50 and good response according to EULAR criteria (table 2). The area under the ROC curve was 0.72 for the final models of predictor identification for ACR50 and good response according to EULAR criteria. Results for all individual predictors analysed are shown in the supplementary material.

**Table 2:** Predictors* of good clinical response to adalimumab at week 12, as defined by ACR50 and good response according to EULAR criteria

Predictors	ACR50 response		Good response according to EULAR criteria	
	OR (95% CI)	p Value	OR (95% CI)	p Value
Sex (male vs female)	1.77 (1.12 to 2.79)	0.014	2.24 (1.39 to 3.59)	<0.001
HAQ-DI (per unit)	0.39 (0.25 to 0.60)	<0.001	0.54 (0.35 to 0.85)	0.007
Systemic glucocorticoids (⩽10 mg/day)†	0.57 (0.35 to 0.92)	0.022	0.53 (0.32 to 0.88)	0.014
Patient’s assessment of pain (per 0–100 mm VAS unit)	1.02 (1.01 to 1.03)	0.003	1.01 (1.00 to 1.03)	0.027
C-reactive protein (per mg/dl)	1.15 (1.01 to 1.30)	0.035	–	–
Swollen joint count (0–76; ⩾5 vs <5)	2.11 (1.13 to 3.94)	0.019	–	–
⩾1 Inflamed large joint†‡	–	–	0.55 (0.34 to 0.89)	0.015
Treatment with SSZ†	–	–	3.11 (1.35 to 7.12)	0.007
Prior DMARD use (⩾2 vs <2)	–	–	1.79 (1.08 to 2.96)	0.024

*For identification of predictors of ACR50 and good responses according to EULAR criteria, the ensemble of all potential factors was investigated by logistic regression with backward elimination (selection level, 5%); †yes vs no; ‡“large joint” could include the shoulder, elbow, knee, or hip.

ACR50, American College of Rheumatology response criteria, 50% improvement; DMARD, disease-modifying antirheumatic drug; EULAR, European League Against Rheumatism; HAQ-DI, Health Assessment Questionnaire Disability Index; OR, odds ratio; PsA, psoriatic arthritis; SSZ, sulfasalazine; VAS, visual analogue scale.

#### Predictors of good clinical response for psoriasis at week 12

Improvement of ⩾3 grades in PGA was experienced by 81 (42%, 13 missing) of the 206 patients with a baseline PGA greater than “mild”. In the evaluation of individual possible baseline predictors, only a PGA greater than “moderate” (crude OR 2.27, 95% CI 1.22 to 4.24; p = 0.011) and a greater PaGA of disease activity (crude OR 1.02, 95% CI 1.00 to 1.03; p = 0.043) were associated with improvement in PGA by ⩾3 grades.

#### Predictors of good clinical response for psoriatic nail disorder at week 12

Improvement in NAPSI score by ⩾50% was experienced by 84 (54.2%, 9 missing) of 164 patients with a baseline NAPSI score ⩾10. In the analysis of individual possible predictors, only lower CRP concentration was associated with ⩾50% improvement in NAPSI score (crude OR 0.79, 95% CI 0.65 to 0.96; p = 0.019).

### Safety

There were 21 serious adverse events, including 4 infections, documented for 18 (4.1%) patients during adalimumab treatment and a 70-day follow-up. Detailed data are summarised in the supplementary material.

## Discussion

The clinical manifestations of PsA in this large study cohort are consistent with the typical pattern of PsA symptoms reported in the literature, and the patient characteristics are representative of those considered eligible for anti-TNF therapy.^1^ ^2^ ^13^ ^14^ After 12 weeks of adalimumab therapy, 74%, 51% and 32% of the patients experienced ACR20, 50 and 70 responses, respectively. These rates are somewhat greater than those found in randomised controlled trials (RCTs) with adalimumab or other TNF antagonists, which may be because the calculated rates in our study were based on observed values.^15^ ^16^ ^17^ Moderate and good responses according to EULAR criteria were achieved by 87% and 61% of patients, respectively, which is similar to the reported rates in pooled data from two RCTs of other anti-TNF agents (etanercept and infliximab).^12^ The safety profile of adalimumab in this 12-week study was consistent with results from RCTs of adalimumab for PsA.^17^ ^18^

Improvement in psoriasis was clinically relevant, with doubling of the baseline percentage of patients with PGA “clear” or “almost clear” skin at week 12 and a median reduction of the target lesion total plaque score by 67%. The results appear to be within the extent of improvement under anti-TNF therapy when other psoriasis assessment tools were used.^15^ ^16^ ^17^

This is the first study that has investigated the effect of adalimumab on nail psoriasis. After the relatively short treatment duration of 12 weeks, the median reduction in NAPSI score was 57%. Clearance of psoriasis of the nails was increasing in those patients who continued adalimumab up to week 20. These results are comparable to those reported for infliximab treatment of nail disorder in patients with PsA.^19^

Our results show that good clinical responses in the various manifestations of PsA (ie, joints, skin and nails) are influenced by various predictive factors. Achievement of ACR50 and a good response according to EULAR criteria was more likely when patients had a lower HAQ-DI score and greater pain. Men had a twofold greater chance of achieving ACR50 and good response according to EULAR criteria compared with women. Systemic glucocorticoid therapy, which is generally not recommended for PsA, decreased the chance of a good response.^1^ The chance of achieving ACR50 was also better for patients with greater CRP concentrations and polyarticular PsA, whereas large joint involvement decreased the chance of a good response according to EULAR criteria. These findings are consistent with a predictor analysis in 69 infliximab-treated patients.^20^

Because several of the potential predictors are components of the response criteria, one might expect that greater baseline values would be associated with greater response. With respect to CRP, for example, it is interesting that lower baseline CRP concentrations predict an ACR50 response for patients with rheumatoid arthritis,^21^ which is in contrast to our findings for patients with PsA. For Bath Ankylosing Spondylitis Disease Activity Index 50% response (BASDAI 50), which does not include CRP, greater baseline CRP concentration predicts a BASDAI 50 response for patients with ankylosing spondylitis.^22^ Thus, CRP appears to be a true biological predictor, and other components of the response criteria may likewise have independent predictive value. Finally, prior use of at least two DMARDs and concomitant treatment with SSZ were associated with the achievement of a good response according to EULAR criteria. Of note, few patients (10.4%) were concomitantly treated with SSZ (either alone or combined with other DMARDs).

The endpoints of a ⩾3-grade improvement in PGA and a ⩾50% improvement in NAPSI score were arbitrarily selected to correspond with ACR50. We found an association between PaGA of disease activity and ⩾3-grade improvement in PGA. Although greater PGA severity at baseline was associated with greater improvement in PGA, this finding appears to be intrinsically caused by the method and should not be considered a real predictor. A ⩾50% improvement in NAPSI score was more likely for patients with lower CRP concentrations at baseline, which is opposite to the impact of CRP concentration on achievement of an ACR50 response.

In conclusion, patients with long-term active PsA experienced clinically important improvement in arthritis, psoriasis and psoriatic nail disorder. Low impairment of physical function (lower HAQ-DI score), greater pain, greater CRP concentration, polyarthritis without inflammation of large joints, prior treatment with >2 DMARDs, current treatment with SSZ but not glucocorticoids and male sex were factors that increased the chance of achieving substantial clinical improvements.
